# Data Descriptor: Pacific Introduced Flora (PaciFLora)

**DOI:** 10.3897/BDJ.9.e67318

**Published:** 2021-07-20

**Authors:** Michael Rudolf Wohlwend, Dylan Craven, Patrick Weigelt, Hanno Seebens, Marten Winter, Holger Kreft, Wayne Dawson, Franz Essl, Mark van Kleunen, Jan Pergl, Petr Pyšek, James Space, Philip Thomas, Tiffany Knight

**Affiliations:** 1 Institute of Biology, Martin Luther University Halle-Wittenberg, Halle (Saale), Germany Institute of Biology, Martin Luther University Halle-Wittenberg Halle (Saale) Germany; 2 German Centre for Integrative Biodiversity Research (iDiv) Halle-Jena-Leipzig, Leipzig, Germany German Centre for Integrative Biodiversity Research (iDiv) Halle-Jena-Leipzig Leipzig Germany; 3 Universidad Mayor, Santiago, Chile Universidad Mayor Santiago Chile; 4 Department of Biodiversity, Macroecology & Biogeography, Faculty of Forest Sciences, University of Göttingen, Göttingen, Germany Department of Biodiversity, Macroecology & Biogeography, Faculty of Forest Sciences, University of Göttingen Göttingen Germany; 5 Senckenberg Biodiversity and Climate Research Centre (SBiK-F), Frankfurt am Main, Germany Senckenberg Biodiversity and Climate Research Centre (SBiK-F) Frankfurt am Main Germany; 6 Durham University, Durham, United Kingdom Durham University Durham United Kingdom; 7 BioInvasions, Global Change, Macroecology-Group, Department of Botany and Biodiversity Research, University Vienna, Vienna, Austria BioInvasions, Global Change, Macroecology-Group, Department of Botany and Biodiversity Research, University Vienna Vienna Austria; 8 Centre for Invasion Biology, Department of Botany and Zoology, Stellenbosch University, Stellenbosch, South Africa Centre for Invasion Biology, Department of Botany and Zoology, Stellenbosch University Stellenbosch South Africa; 9 University of Konstanz, Konstanz, Germany University of Konstanz Konstanz Germany; 10 Czech Academy of Sciences, Institute of Botany, Department of Invasion Ecology, Průhonice, Czech Republic Czech Academy of Sciences, Institute of Botany, Department of Invasion Ecology Průhonice Czech Republic; 11 Institute of Botany, Průhonice, Czech Republic Institute of Botany Průhonice Czech Republic; 12 Academy of Sciences of the Czech Republic, Pruhonice, Czech Republic Academy of Sciences of the Czech Republic Pruhonice Czech Republic; 13 Pacific Southwest Research Station, USDA Forest Service (ret.), Sun Lakes, United States of America Pacific Southwest Research Station, USDA Forest Service (ret.) Sun Lakes United States of America; 14 Hawaiian Ecosystems at Risk project, Carrboro, United States of America Hawaiian Ecosystems at Risk project Carrboro United States of America; 15 Martin-Luther-Universität Halle-Wittenberg, Leipzig, Germany Martin-Luther-Universität Halle-Wittenberg Leipzig Germany

**Keywords:** Island Biogeography, naturalised species, Pacific Ocean, plant invasion, species database

## Abstract

**Background:**

The Pacific Region has the highest density of naturalised plant species worldwide, which makes it an important area for research on the ecology, evolution and biogeography of biological invasions. While different data sources on naturalised plant species exist for the Pacific, there is no taxonomically and spatially harmonised database available for different subsets of species and islands. A comprehensive, accessible database containing the distribution of naturalised vascular plant species in the Pacific will enable new basic and applied research for researchers and will be an important information source for practitioners working in the Region.

**New information:**

Here, we present PacIFlora, an updated and taxonomically standardised list of naturalised species, their unified nativeness, cultivation and invasive status and their distribution across the Pacific Ocean, including harmonised location denoination. This list is based on the two largest databases on naturalised plants for the Region, specifically the Pacific Island Ecosystems at Risk (PIER) and the Global Naturalised Alien Flora (GloNAF) databases. We provide an outlook for how this database can contribute to numerous research questions and conservation efforts.

## Introduction

The Pacific Ocean covers a large area and contains over 25,000 islands (National Oceanic and Atmospheric Administration & Western Pacific Regional Fishery Management Council 2009). A rich endemic flora has evolved in the Pacific, which is now threatened by, amongst other drivers, an increasing number of naturalised plant species (Seebens et al. 2017, van Kleunen et al. 2019). The Pacific Ocean is unique, with vast areas of ocean stretching between thousands of islands that create substantial barriers to the natural dispersal of plant species. It is also unique in its relatively recent colonialisation history through Polynesian and later, European settlers (Matisoo-Smith and Robinson 2004) and the large socio-economic differences that exist between island groups of different geological origin (*Seidel and Lal 2010*). However, human-mediated dispersal has resulted in many islands being inhabited by naturalised plant species, defined as alien plant species that maintain self-sustaining populations without human intervention (Richardson et al. 2000). Understanding naturalisation is facilitated by detailed information about introduction and establishment processes. Although some alien plants are introduced accidentally (e.g. stowaways, contaminated seeds), most of them are deliberately introduced for cultivation (e.g. for ornamental or other economic uses; Hulme et al. 2008, van Kleunen et al. 2020). The extent to which plant species escape cultivation and become naturalised will vary in space and time, so that a single species can be considered cultivated in one location and naturalised in another location, even in close proximity. Likewise, the shift of a plant species from naturalised to invasive, that is when species harm the environment or humans (sensu Richardson et al. 2000, Blackburn et al. 2011), will also vary in space and time. Information on a species’ invasion status in one location may be useful for the development and implementation of measures designed to mitigate its impacts or prevent invasions in other locations across the region. Finally, because of the vastness of the Pacific, intra-Pacific naturalisations occur, i.e. some species are categorised as naturalised on some islands, but native on others.

To address research questions in an objective and accessible way, databases are required that contain occurrences (presences) of naturalised plant species and harmonised region information that span the whole Pacific Region, while also being interoperable with other databases (e.g. origin, BIEN, TRY and GIFT; Kattge et al. 2011, Weigelt et al. 2019, Chamberlain and Bartomeus 2020, Maitner 2020). Additional features, such as cultivation and invasive status, can extend the range of applicability. The Global Naturalised Alien Flora (GloNAF) and the Pacific Island Ecosystems at Risk (PIER) databases meet these criteria. While these databases provide unique information on species’ characteristics (e.g. cultivation, invasive status), they also overlap in information for many naturalised species and locations. However, even when the information overlaps between the databases, there are sometimes different names or spellings for the same islands or species and there is also variation between the databases in the quality and method of evaluating species invasion status. In addition, information is available inconsistently at different spatial scales, namely at island group and individual island level. These sources of data variation in the databases present a challenge to the direct combination and use of the databases in a single study.

**Figure 1.**
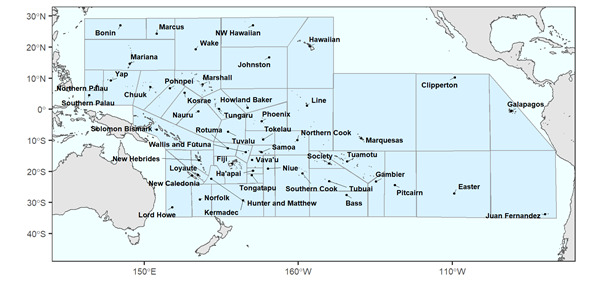
Map showing the boundaries of island groups in the Pacific used in this database. Underlying map: World Coastline for R, based on data from Natural Earth. Polygons surrounding island groups are designed to include all islands in the group using straight lines and, thus, these lines do not correspond to any political border.

Here, we present PacIFlora, a consolidated database on naturalised plant species on Pacific islands which overcomes the challenges posed by combining two large databases. By merging, harmonising and standardising information on naturalised species on Pacific islands from GloNAF and PIER, we created this new database reporting the presence of naturalised plant species on each island or island group. We also categorised the islands with available data into sociogeographic groups, as this is useful for many invasion science research questions (Fig. 1, see also Wohlwend et al. 2021). GloNAF was initiated in 2011 and launched in 2015 as a worldwide database of naturalised plant occurrences in mostly geopolitical regions (Pyek et al. 2017, van Kleunen et al. 2015, van Kleunen et al. 2019). PIER is a project of the Institute of Pacific Islands Forestry (USDA Forest Service) initiated in 1997 to compile and disseminate reference information on alien plant species of known or potential threat to Pacific island ecosystems (http://www.hear.org/pier/). These two databases are, to our knowledge, the only ones covering the entire Pacific, which was important for us to not further artificially increase sampling effort differences amongst regions.

We structured our data and R code in a way that makes PacIFlora easy to combine with other databases. We also provide our R code to facilitate the integration of additional data, in case, for example, a user of our database wants to focus on a smaller part of the Pacific Region or integrate additional data.

**Figure 2.**
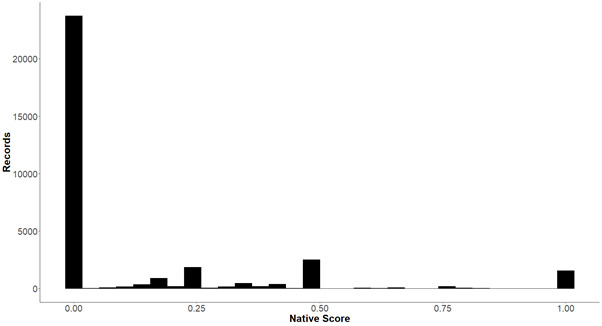
Total number of records in categories of native status. Nativeness Score indicates naturalisation certainty, i.e. 0 indicates records that are certainly naturalised and 1 indicates records that are certainly native, respectively. Intermediate values indicate uncertainty in the native status. See main text for detailed clarification.

Accepted plant species scientific names were identified using the recently-published Leipzig Catalogue of Vascular Plants using the original names (LCVP, Freiberg et al. 2020). In total, this resulted in 33301 unique records, including 3963 species distributed over 482 islands aggregated in 50 island groups. A total of 125 records from 34 unique original species names could not be assigned to an accepted species name by the algorithm and were included as “NA”. Manual matching is possible for some of them, but we abstained from this as we wanted to exclude all subjectivity from our side. A total of 847 records lack island level information and, as a result, the species x island matrix has fewer species then the species x island group matrix. The output table of PacIFlora includes plant family, plant order invasion status (if a species is currently evaluated as harmful on a certain island), native status (how likely a species is to be considered native on an island), cultivation status (how likely a species only exists as a cultivar on an island) and the name and coordinates of each island. We show the relative frequency of different categories of native status in Fig. 2.

**Figure 3.**
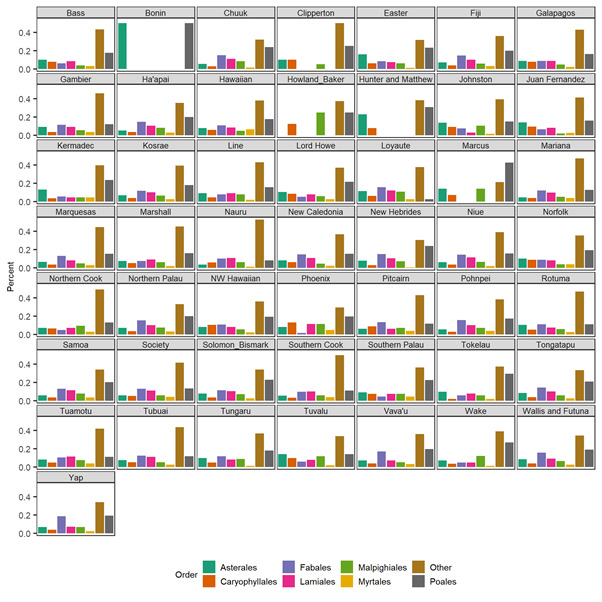
Proportional representation of naturalised plant species in the seven most common orders for each island group.

In addition, we provide a phylogeny of the naturalised plant species in PacIFlora by pruning the comprehensive supertree by Smith and Brown 2018) to all species it has in common with PacIflora (3150) and adding the remaining (813) via a congeneric merge resulting in some polytomies. All genera where found in the supertree. Fifty-four orders of naturalised plants are present in the Pacific. Most naturalised plant species in PacIFlora belong to the orders Poales, Fabales, Lamiales, Asterales, Caryophyllales, Myrtales and Malpighiales (in descending order), but the relative representation of these orders varies across island groups (Fig. 3).

This database can be used to address a wide variety of research questions and for management applications, for example, by combining it with different datasources on environmental drivers (Wohlwend et al. 2021), taxonomic or trait information, native flora information or data on dispersal pathways. There is also potential to use PacIFlora for invasion forecasting and species distribution modelling.

## General description

### Purpose

This dataset can be used for research on a wide variety of questions, including: (1) the study of patterns of richness and composition of naturalised plants in the Pacific and the roles of anthropogenic and biogeographic drivers (Wohlwend et al. 2021); (2) the study of patterns of taxonomic and phylogenetic composition of naturalised plants in the Pacific compared to other regions of the world; (3) the development of forecasting tools to identify naturalised species that are present in the Pacific and are likely to expand their ranges to new island groups; (4) for comparing patterns of native and naturalised species richness and composition to test whether islands poor in native species are more vulnerable to invasion and (5) for identifying mechanisms that determine the range of naturalised species by combining this dataset with information about functional traits (e.g. Kattge et al. 2011:TRY www.try-db.org) and common introduction pathways.

This dataset represents a second step (after PIER and GloNAF) towards the development of a comprehensive list of the presence and status of naturalised plant species in the Pacific Region. Important next steps involve validating and updating these data in strong collaboration with local experts from each island group. For example, naturalised data exist for 488 of the > 25000 islands in the Pacific. It remains to be validated whether the remaining islands in the Pacific really do not have established naturalised plant species (e.g. the numerous tiny atolls) or whether local information about naturalised plant presence was not included in the two region-spanning sources and, thus, in PacIFlora. We hope that PacIFlora can serve as a foundation for local organisations in the Pacific that can be updated and extended in the future. The authors provide their full support for the application, validation and extension of PacIFlora. Main contact persons for this are Michael Wohlwend (application), Mark van Kleunen (validation and extension, GloNAF) and Philip Thomas (validation and extension, PIER).

We note that the results in Wohlwend et al. (2021) used a subset of the records in PacIFlora (e.g. excluding all cultivated records for most analyses) and considered data aggregated by island group. PacIFlora aims to provide more comprehensive resources that can be used for other purposes than those that were the focus of Wohlwend et al. (2021), but information presented in this publication can give insights into the data.

## Project description

### Design description

To create a matrix of species presences on islands and island groups, we used raw data from GloNAF version 1.1 and raw data from PIER (updated 2 June 2018). Both PIER and GloNAF list their sources for all records of a naturalised species on an island.

We harmonised species names using the LCVP (Freiberg et al. 2020) and the associated R-package ‘lcvplants’ (https://github.com/idiv-biodiversity/LCVP). Subspecies and varieties were aggregated to the binomial level, which we refer to as “species” level for simplicity. If hybrid taxa were not recorded in the LCVP database, it was pooled with the first parent species, affecting 20 species. Forty species names were identified by the LCVP as synonyms for more than one possible species. In these cases, we chose the first species name provided by the LCVP as the assigned name to ensure reproducibility. Twenty species could not be linked to an accepted name by the LCVP with certainty and were assigned ‘NA’ values for species name, family and order. We kept these unassignable species in the list format of PacIFlora to allow for future name resolution.

All calculations were performed and graphs were created using R (version 4.0.3, R Core Team 2020). Maps were created using R and the packages ‘ggplot2’ (Wickham 2016), ‘ggtree’ (Yu 2020) and ‘rnaturalearth’ (South 2017) for visualisation. We created background polygons for island group association using QGis 3.12.3 (QGIS Development Team 2021). Matrix aggregation was performed using the fuzzySim package in R (Barbosa 2015).

PacIFlora includes the following columns: ID, Species, Island, Island group, Family, Order, Native, Cultivated, Source, Original_Name, Invasion. **ID** provides a unique number. **Species** is the accepted name of the species based on the LCVP. A total of 3963 species were recorded. **Island** is the location where the species is present. Islands often have many names or different spellings. We used web research and other information in our sources to identify synonyms and chose one name from the sources (usually the most commonly used name). Island refers to the smallest available unit of reference and is, therefore, sometimes used for several unnamed islets of an atoll in close proximity which are not or only sometimes (tidally) connected by land. A total of 488 islands where recorded. **IslandGroup** is a group name assigned by us. The inclusion of island groupings is useful for many types of research questions, as a complete species list at a broader spatial scale decreases problems of data deficiency for individual islands in an archipelago. Our groups are largely based on political borders, such as municipalities or states. If political borders did not reflect geographic borders, we used distance between islands and ocean trenches to assign each island to one of 50 island groups. Island group aggregation is visualised in Fig. 1. We excluded 146 records that could not be linked to any island group. This particular grouping is useful for questions related to the influence of dispersal barriers on biological invasions, as distance creates a natural barrier and political borders are known to influence dispersal via human imports (either intentional or accidental). However, islands also vary in age, size and geomorphology and, thus, we make it possible to regroup the islands in our database into formats that might be better suited for other research questions (e.g. on establishment barriers). **Family** is the plant family and **Order** is the plant order. **Native** status indicates the certainty if the species is native at the given location. While all of the species in PacIFlora are naturalised in at least one location in the Pacific, some species might be native in other locations of the Pacific. We assigned numerical values, indicating certainty of native status on each specific island between 0 (unanimously described as naturalised on the specific island) and 1 (unanimously described as native on the specific island) to the categorical classes defined in GloNAF and PIER, which were averaged if there was no agreement amongst the sources. There were 36 species with only “native” records, which were excluded. The vast majority of records (94%) are not described as likely native. A total of 255 species were described at least once as likely native (Fig. 2). **Cultivated** provides information on whether the species is classified as being only cultivated on the island or island group. Cultivated values of 1 define species that are only known to exist in horticultural plantings at the location, whereas those clearly described as naturalised by any source at the location are given a value of zero. A value of 0.5 indicates that there is no information available documenting whether the species is either cultivated or naturalised at the location. There were 40 species with only “cultivated” records, which were excluded. A total of 19,944 records were described as not cultivated, 9150 had no information on cultivation status and 4207 were described as cultivated. A total of 630 (16%) species were described as cultivated at least once, which also means that those species escaped cultivation at least once. Cultivated and native scores were determined differently since a species can be both cultivated and naturalised on an island, but not native and alien. **Database** indicates if the record was present in GloNAF (glon), PIER (pier) or both (glon_pier). **Orignal_Name** shows the species name prior to standardisation. **Invasive** status is a column indicating invasive status of the species on a given island, with “1” meaning unanimously described as invasive in this location, “0” meaning unanimously described as not invasive. The value in this column was achieved by forming a mean of the evaluation of all records for a particular species island combination, why this column should be handled with care, as there was no information if one occurrence was evaluated differently by different authors or if two different occurrences on one island were evaluated differently. In total, 15,713 records where described as likely invasive, including 1550 species. **Source** provides the original reference as listed in GloNAF and PIER. Only one reference is provided for each record and additional references can be accessed via the **Source_ID** column, which lists the IDs of all references listing this record. References for the IDs can be found in Suppl. material 3. Most records have only one record, but a record can have as many as eighteen references (e.g. due to voucher specimens). An overview of the 22 most frequently used sources can be seen in Table 1. **Latitude** and **Longitude** give the geographical coordinates of the island centroid in decimal degrees, which were taken from the Global Inventory of Floras and Traits (GIFT) database (Weigelt et al. 2013, Weigelt et al. 2019). In total, 84 islands could not be connected to a unique ID in GIFT; coordinates for these were taken from Google Maps (Google 2020). All coordinates are provided in WGS84.

We present our database in three formats:

(1) PacIFlora - Full list format of all records (species x island, including records with no information on island, but just island group level and records that could not be identified by the LCVP), Suppl. material 2.

Additionally, you can find the following files:

(2) An island × species matrix, excluding records that were missing information. Specifically, this list does not include data that have no island information or species that could not be identified by the LCVP (Dryad only).

(3) An aggregated island group × species matrix (Dryad only).

(4) A table to access reference IDs, Suppl. material 3.

(5) List format of PacIFLora on island level, excluding all records with no information on island, but just island group level and records that could not be identified by the LCVP (Dryad only).

(6) List format of PacIFLora on island group level, excluding all records that could not be identified by the LCVP and providing aggregated values for naturalisation, cultivation and invasion status (Dryad only).

Both data matrices (2 and 3) are included to provide an easy-to-use format for research and conservation applications. When using these matrices, be aware that they include all records (e.g. including cultivated species for some records). We provide our full R code used for aggregation, starting from GloNAF and PIER raw data, which allows, for example, for the generation of personalised subsets.

To create a phylogeny for the naturalised plant species in the Pacific, we pruned the supertree by Smith and Brown (2018). Species names in PacIFlora and in this supertree were first harmonised using the LCVP (Suppl. material 1). Focal species which were missing from this supertree were grafted onto it at the genus level using the function congeneric.merge in the “pez” package of R (Pearse et al. 2015). We summarise the spatial variation of plant order composition on island groups in a bar chart, showing the proportional representation of species in the seven overall most common (measured in species/family) plant orders (Figs 1, 3).

Our R code allows a complete workflow from the publicly available PIER and GloNAF data to the final species x island matrix. All codes used to unify and aggregate the data are provided in the R programming language and is open access via github (https://github.com/MichaelWohlwend42/PacIFlora.git). We provide code to merge new data with PacIFlora in a standardised manner, using universally applicable harmonisation functions for species and islands. The data underpinning the analysis reported in this paper are deposited in the Dryad Data Repository at https://datadryad.org/stash/dataset/doi: 10.5061/dryad.qfttdz0hd, as well as partly in the supplementary material and on the abovementioned GitHub Page.

## Geographic coverage

### Description

PaciFlora covers all islands with available data in the Pacific Ocean. Only oceanic islands between 40°N and 40°S are included as our focus was on (sub-)tropical islands. Larger landmasses, such as Japan, New Zealand the Philippines and Papua New-Guinea, as well as all islands on the Japanese, Pacific American or Australian coasts, were excluded. So, this database focuses on (sub-)tropical islands that are isolated from larger landmasses.

## Usage licence

### Usage licence

Creative Commons Public Domain Waiver (CC-Zero)

## Data resources

### Data package title

Pacific Introduced Flora (PaciFLora)

### Number of data sets

1

### Data set 1.

#### Data set name

PaciFlora

#### Number of columns

14

#### 

**Data set 1.** 

Column label	Column description
Species	Simple species name.
Island	island name.
IslandGroup	assigned island group name.
Native	Standardised native score ranging from 0 (unanimously described as not native) to 1 (unanimously described as native) in given location.
Cultivated	Standardised cultivation score ranging from 0 (unanimously described as not cultivated) to 1 (unanimously described as cultivated) in given location.
Family	plant family.
Order	plant order.
Database	Origin GloNAF, PIER or both (glonpier).
Invasive	Standardised invasive score ranging from 0 (unanimously described as not invasive) to 1 (unanimously described as invasive) in given location.
Orginal_name	pre-harmonisation species name.
Latitude	latitude of island (mercator).
Longitude	longitude of island (mercator).
Source	Literature cited for this entry in the raw data.
Source_ID	Full list of references provided for this occurrence, which can be referenced using the attached list.

## Supplementary Material

0691724C-DBCA-5DBF-B7A3-2AD8622F0885 10.3897/BDJ.9.e67318.suppl1 Supplementary material 1 PacIFlora Phylogeny Data type Phylogeny File: oo_523064.tre https://binary.pensoft.net/file/523064 Smith & Brown, harmonised, pruned and extended by Michael R Wohlwend

7B3B4B79-5D9B-5817-8817-3E6DCB192AB2 10.3897/BDJ.9.e67318.suppl2 Supplementary material 2 PacIFlora Data type List, Species, Location, Additonal Information Brief description Complete Datasbase in list form, as it will be uploaded to zenodo and GitHub. File: oo_551809.txt https://binary.pensoft.net/file/551809 Us

A6546781-43B5-599D-B9CD-DAD07B44A3FE 10.3897/BDJ.9.e67318.suppl3 Supplementary material 3 Reference Table Data type Sources and Source IDs Brief description Allows for linking additional source ids to the actual source File: oo_551767.txt https://binary.pensoft.net/file/551767 Michael Wohlwend

## Figures and Tables

**Table 1.** Table 1: Most frequent sources used in PacIFlora. We note that some of the sources might provide overlapping information on naturalised plant occurrences. Records refers to species × island occurrences. Sources are sorted in decreasing order, based on the number of records each source provide. The names of the three island groups with the most records from each source are displayed.

Source	Records	Most recorded island groups
Gargominy et al. 2018	9126	New Caledonia, Clipperton
Imada 2019	5104	Hawaiian, NW Hawaiian
Florence et al. 2013	4119	Society, Marquesas, Tubuai
Fosberg et al. 1979	2780	Mariana, Northern Palau, Yap
Wagner et al. 1999	2600	Hawaiian, NW Hawaiian, Solomon_Bismark
Raulerson 2006	1982	Mariana
Charles Darwin Foundation 2008	1601	Galapagos, Solomon_Bismark
Florence et al. 2007	1600	Society, Marquesas, Tubuai
Fosberg et al. 1987	1467	Mariana, Northern Palau, Chuuk
McCormack 2007 McCormack 2013	1170	Southern Cook, Northern Cook, Chuuk
McCormack 2007	1076	Southern Cook, Northern Cook
MacKee 1994	1054	New Caledonia, Loyaute, Hunter and Matthew
Lorence and Wagner 2013	1042	Marquesas
Wagner and Lorence 2002	1020	Marquesas
Space et al. 2003	881	Northern Palau, Southern Palau
Welsh 1998	781	Society, Bass, Tuamotu
Guézou et al. 2014	775	Galapagos
Invasive Species Specialist Group ISSG. 2019	635	Tongatapu, Nauru, Easter
Whistler 1998	614	Samoa, Tokelau
Florence 2004	599	Society Marquesas, Tuamotu
Swarbrick 1997	574	Solomon_Bismark, New Caledonia, New Hebrides
Yuncker 1959	519	Tongatapu, Vava'u, Ha'apai
